# Intracranial pressure monitoring following traumatic brain injury: evaluation of indications, complications, and significance of follow-up imaging—an exploratory, retrospective study of consecutive patients at a level I trauma center

**DOI:** 10.1007/s00068-020-01570-3

**Published:** 2020-12-22

**Authors:** Alexander Bumberger, Tomas Braunsteiner, Johannes Leitgeb, Thomas Haider

**Affiliations:** Department of Orthopedics and Trauma Surgery, Medical University of Vienna, Vienna, Austria

**Keywords:** Traumatic brain injury, Intracranial pressure monitoring, Cranial computed tomography, Follow-up, Neurotrauma, Intensive care

## Abstract

**Background:**

Measurement of intracranial pressure (ICP) is an essential part of clinical management of severe traumatic brain injury (TBI). However, clinical utility and impact on clinical outcome of ICP monitoring remain controversial. Follow-up imaging using cranial computed tomography (CCT) is commonly performed in these patients. This retrospective cohort study reports on complication rates of ICP measurement in severe TBI patients, as well as on findings and clinical consequences of follow-up CCT.

**Methods:**

We performed a retrospective clinical chart review of severe TBI patients with invasive ICP measurement treated at an urban level I trauma center between January 2007 and September 2017.

**Results:**

Clinical records of 213 patients were analyzed. The mean Glasgow Coma Scale (GCS) on admission was 6 with an intra-hospital mortality of 20.7%. Overall, complications in 12 patients (5.6%) related to the invasive ICP-measurement were recorded of which 5 necessitated surgical intervention. Follow-up CCT scans were performed in 192 patients (89.7%). Indications for follow-up CCTs included routine imaging without clinical deterioration (*n* = 137, 64.3%), and increased ICP values and/or clinical deterioration (*n* = 55, 25.8%). Follow-up imaging based on clinical deterioration and increased ICP values were associated with significantly increased likelihoods of worsening of CCT findings compared to routinely performed CCT scans with an odds ratio of 5.524 (95% CI 1.625–18.773) and 6.977 (95% CI 3.262–14.926), respectively. Readings of follow-up CCT imaging resulted in subsequent surgical intervention in six patients (3.1%).

**Conclusions:**

Invasive ICP-monitoring in severe TBI patients was safe in our study population with an acceptable complication rate. We found a high number of follow-up CCT. Our results indicate that CCT imaging in patients with invasive ICP monitoring should only be considered in patients with elevated ICP values and/or clinical deterioration.

## Introduction

According to a recent meta-analysis, the overall incidence of traumatic brain injury (TBI) in Europe is about 262/100,000 with considerable heterogeneity between the included studies [1]. While the incidence of traffic-related TBI is decreasing in high-income countries, brain injuries in elderly, caused mainly by falls, increased due to demographic development and higher life expectancy. Comorbidities and anticoagulation therapy frequently complicate management of these patients. Globally, the overall incidence of TBI is increasing primarily due to rising motorized traffic in developing countries [2].

In the context of TBI, the generally recognized Monro–Kellie doctrine and the equation cerebral perfusion pressure (CPP) = mean arterial pressure (MAP)—intracranial pressure (ICP), as proposed by Miller et al. [3, 4], has shaped our understanding of cerebral perfusion and its correlation with MAP and ICP. From this insight, the concept of monitoring ICP in TBI patients was deducted. Current guidelines of the Brain Trauma Foundation recommend ICP monitoring for patients with severe TBI (GCS 3–8 after resuscitation) [5]. There are two options for invasive ICP measuring, including intraparenchymal probes and intraventricular catheters. The latter is considered the gold standard as it measures the global ICP, while also providing therapeutic cerebrospinal fluid drainage if necessary [6]. Multimodal monitoring including measurement of intracerebral metabolic parameters and its utility in TBI patients has also been scrutinized in recent studies [7–9]. Due to a lack of large randomized controlled trials (RCT), there is an ongoing discussion as to whether invasive ICP monitoring can decrease mortality in severe TBI [10–12]. The only two RCTs we are aware of, include a limited number of patients and do not support the hypothesized lower mortality in ICP-monitored TBI patients [13, 14]. While ICP monitoring is also associated with catheter-related complications, such as focal hemorrhage and local infection in about 6 percent of the interventions [15], it is still considered an essential monitoring tool in sedated patients following severe TBI. The gold standard of imaging in these patients is cranial computer tomography (CCT) which further guides the treatment strategy in these patients. Follow-up CCT in intubated patients is required to monitor potential dynamic changes of intracranial hemorrhage and swelling. In intubated patients, neurological examination cannot be performed, prompting frequent follow-up CCT examinations which require patient transportation and positioning, both potentially increasing ICP [16, 17]. Possible compartmentalization and the possibility of inadequate pressure monitoring often lead to follow-up CCTs also in patients with implanted invasive ICP monitoring. However, there is no report available showing frequency and findings of follow-up CCT in ICP-monitored patients following severe brain trauma. Also, studies evaluating rates and types of complications of invasive ICP monitoring in these patients are scarce. Therefore, the aims of this study were threefold: (1) evaluating the short-term outcome in patients with invasive ICP-measurement following severe TBI, (2) analyze rates and findings of follow-up CCTs and their impact on clinical decision making, and (3) report on complications associated with invasive ICP monitoring in these patients.

## Methods

Institutional review board approval by the local ethics committee was obtained (approval number 2086/2018). A systematic database search for patients who received an ICP-measuring probe between January 2007 and September 2017 at our urban level I trauma center was conducted. Data acquisition was performed by reviewing electronic health records of each patient. Extracted data included patient gender, age, date of admission, mechanism of trauma, Glasgow coma scale (GCS) on admission, CCT reports, surgery reports including probe implantation, trepanation, craniotomy/craniectomy and potential revisions, as well as length of hospitalization, and comorbidities/additional injuries. Patients were excluded in case of poor documentation or missing medical records. The Injury Severity Score (ISS) was calculated in each case and patients were stratified as non-polytraumatized or polytraumatized at a cut-off for polytraumatized patients of ISS ≥16.

Parenchymal probes distributed by Codman^®^ and Raumedic^®^ were used during the observational period. Indications for ICP monitoring were based on findings from the initial CCT (listed in Table 1). Implantation of the probe was performed following intubation in the operating room or in the trauma bay according to manufacturer’s instructions. In cases of cranial decompression surgery implantation of the probe was performed on the contralateral side. Otherwise the side of more pronounced injury was selected for ICP monitoring. Then, patients were transferred to the intensive care unit (ICU) and treated according to the local TBI protocol including elevated upper body, maintaining of cerebral perfusion-optimized blood pressure, adequate oxygenation, normocapnia, and minimized handling and positioning of the patient among others. Probe removal was indicated based on individual clinical findings including neurological status, local soft tissue presentation, and CCT findings.

**Table 1:** Patient demographics of study population

	*N*	%
Sex		
Male	160	75.1
Female	53	24.9
Age		
Mean (range)	44.0 (1–92)	
Severity of injury		
ISS ≤ 15	166	80.2
ISS ≥ 16	41	19.8
Findings of initial CCT		
EDH	40	18.7
SDH	109	50.9
SAH	101	47.2
ICH	11	5.1
Edema	72	33.6
ML-Shift	93	43.5
Fracture	121	56.5
Mechanism of trauma		
Falls	96	45.1
Traffic accident	68	31.9
Fall from standing	45	21.1
Fall from height	29	13.6
Fall over staircases	22	10.3
Violence/abuse	9	4.2
Sports	6	2.8
Penetrating injury	2	0.9
Other	28	13.1
Initial ype of Trepanation/Craniotomy		
Burr hole	102	46.9
Craniectomy	95	44.6
Craniotomy	16	7.5
Mortality		
Deceased	46	21.6
Survived	167	78.4
Duration of ICU stay (d)		
Median (IQR)	32.0 (15–63)	
Interval postop. CCT to f/u CCT (h)		
Median (IQR)	24.1 (7–60)	
*N* of CCT/patient		
Median (IQR)	6.0 (4–9)	
GCS on admission		
Median (Range)	3 (3–15)	
GOS at transfer		
Median (Range)	3 (1–5)	

*ISS* Injury Severity Score, *TBI* traumatic brain injury, *EDH* epidural hematoma, *SDH* subdural hematoma, *SAH* subarachnoid hemorrhage, *ICH* intracerebral hemorrhage, *ML-Shift* midline-shift, *ICU* intensive care unit, *d* days, *f/u* follow-up, *CCT* cranial computed tomography, *GCS* Glasgow Coma Scale, *GOS* Glasgow Outcome Scale

Initial CCT scans as well as the first two follow-up CCT scans were reviewed regarding relevant features indicating TBI (intracranial hemorrhage, fracture, edema, midline-shift), and their spatiotemporal characteristics. As follow-up scans were routinely performed after implantation of the ICP probe, the following (third) CCT scan was considered the first “true” follow-up scan. Indications for these follow-up scans were extracted from clinical records and stratified as “routine follow-up” (without clinical deterioration or elevated ICP), “follow-up due to clinical deterioration” (new onset of anisocoria, cardiovascular decompensation etc.), and “follow-up due to elevated ICP” (> 20 mmHg).

In terms of outcome measures, the physical and neurological constitution of patients was extracted from letters of discharge and then quantified according to the Glasgow Outcome Scale (GOS) [18]. In case of decease (GOS 1), time and cause of death was recorded.

### Statistical analysis

A descriptive analysis focusing on the indications and bleeding progression of follow-up CCT scans based on the available clinical information and proportions were calculated. Distribution of variables was assessed with the Shapiro–Wilk test and graphically confirmed using histograms. Characteristics of given variables are provided in the results section. Interquartile range (IQR) is provided were appropriate. Chi-square test was performed to compare progression of CCT findings among the three indications for follow-up CCT (routine, elevated ICP, and clinical deterioration). Also, odds ratios (OR) with 95% confidence interval (CI) were calculated. A *p* value < 0.05 was considered statistically significant. All calculations were performed using SPSS (Version 25, IBM, USA).

## Results

### Demographics

A total of 214 patients at a mean age of 44 (range 1–92) years received an ICP-measuring probe at our department between January 2007 and September 2017. One patient had to be excluded due to missing trauma records. One-hundred-and-sixty patients were male (75.1%) and 53 were female (24.9%). Forty-one patients (19.8%) were polytraumatized (ISS ≥ 16) and 166 (80.2%) were non-polytraumatized (ISS ≤ 15). Falls were the most common mechanism of injury, accounting for 45.1% of all reported cases, followed by traffic accidents (31.9%) (Table 1) At the time of admission to hospital, the mean GCS score was 6.3 (median 3). The intra-hospital mortality rate within the study population was 20.7% (*n* = 44). All patients underwent primary surgical intervention including isolated implantation of the ICP probe or in combination with a burr hole trepanation in 102 patients (47.9%), with additional craniectomy in 95 patients (44.6%), and in 16 patients (7.5%) with craniotomy.

### Cranial computed tomography (CCT) and clinical implications

**Fig. 1:**
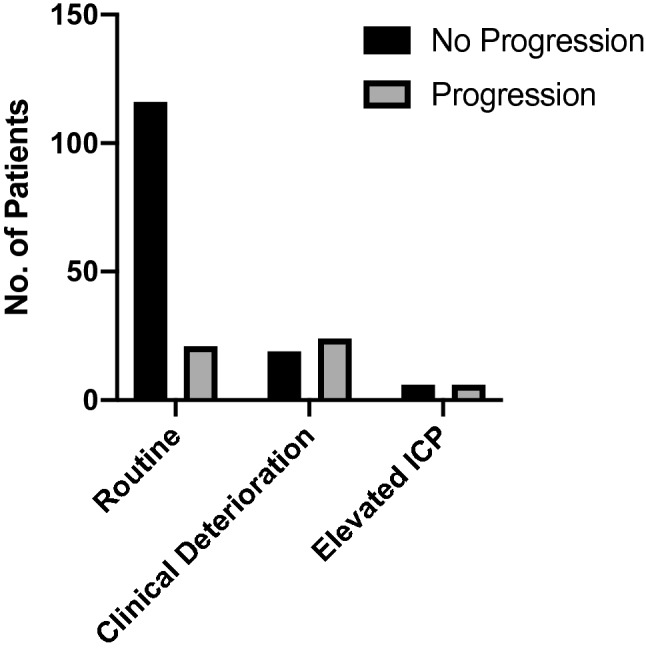
Side-by-side comparison of progression and non-progression of cranial computed tomography (CCT) findings grouped by indication of follow-up CCT. *ICP* intracranial pressure

Overall, follow-up CCTs were performed in 192 patients. Prevalence of critical features in the initial CCT scan such as intracranial hemorrhage, fracture, edema and midline-shift are displayed in Table 1. Seven CCT scans per patient were performed during hospitalization on average. The majority of follow-up CCT scans (*n* = 137, 64.3%) were routine follow-up scans. One in four follow-up scans (*n* = 55, 25.8%) was performed due to either clinical deterioration or elevated ICP. Twelve patients (5.6%) deceased before a follow-up could be performed, in one case, no follow-up was performed because the patient was a child, the remainder (*n* = 8, 3.7%) had poor documentation with no explicit CCT indication. The rate of bleeding progression on routinely performed follow-up CCTs was 16.1% (22/137). In case of clinical deterioration or elevated ICP the proportion of progression of the initial CCT findings (i.e. bleeding progression, additional intracranial hemorrhage and/or increase of brain edema) was 50.0% (6/12) and 55.8% (24/43), respectively. Follow-up scans performed due to clinical deterioration or elevated ICP were each significantly associated with aggravation of CCT findings as compared to routine follow-up CCTs (clinical deterioration: *x*^2^(1) = 8.94, *p* < 0.01, OR = 5.52, 95% CI 1.63–18.77; elevated ICP: *x*^2^(1) = 28.61, *p* < 0.001, OR = 6.98, 95% CI 3.26–14.93) (Table 2). Figure 1 demonstrates the frequency of bleeding progression according to the indication of CCT.

**Table 2:** Odds ratio (OR) for aggravation of cranial computed tomography (CCT) findings compared to routinely performed follow-up CCT

	OR	95% CI	*p* value
Clinical deterioration	5.52	1.63–18.77	< 0.01
Elevated ICP	6.98	3.26–14.93	< 0.001

*CI* confidence interval, *ICP* intracranial pressure

A subgroup analysis was performed including patients who received primary craniotomy versus patients who did not. There was no significant difference regarding the associations between CCT indications and bleeding progression.

On routine follow-up CCT, 22 cases of aggravation of imaging findings were detected (16.1%). These findings did not yield any clinical implication in 68.2% (15/22) of these patients and 94.9% of all patients with routine follow-up CCTs (7/137), respectively. The most common clinical consequence was a delay of antithrombotic therapy in 18% (4/22) of the cases. There was one case of surgical intervention with removal of a newly detected epidural hematoma.

On 55 follow-up CCTs performed due to either clinical deterioration or elevated ICP, 30 cases of aggravated initial imaging findings were detected (30/55, 54.5%). In the majority of these patients (76.7%, 23/30), imaging dynamic did not result in any clinical implication. The most common clinical consequence was a craniectomy in 10% (3/30) of the cases.

Of all patients who received follow-up CCT imaging (*n* = 192), a total of six patients (3.1%) underwent surgical intervention subsequently.

### Catheter-associated complications

There were 12 (5.6%) complications associated with the ICP-probe (Table 3). Breakage, failure and dislocation were considered technical failures, whereas bleeding adjacent to the probe and infection were considered surgical complications. Surgical revision was performed in five cases as a consequence of these complications. Altogether, there were no severe adverse events (major bleeding, death) directly related to the probe or its implantation and no catheter-associated infections.

**Table 3:** Complications associated with intraparenchymal probes

	*N*	Subtype/consequence
Technical failures		
Probe breakage	1	Tip of probe broken off/revision
Probe dislocation	4	1 revision, 3 removals
Probe failure (not specified)	1	Removal
Perioperative complications		
Bleeding at probe site	6	3 subgaleal hematoma, 1 bleeding alongside probe, 2 others/revision
Total	12	No severe adverse events directly related to probe

### Clinical outcome

There were no significant associations between the prevalence of defined CCT scan characteristics (intracranial hemorrhage, fracture, edema and midline-shift) and patient mortality (*p* > 0.05). The overall in-hospital mortality in the analyzed study population was 21.6%. Twelve patients (5.6%) deceased within the first 48 h from admission. Patients were discharged to rehabilitation or a secondary care facility after a median stay of 32 days (IQR: 15–63, range 0–230) and a median GOS of 3 (range 2–5) at transfer.

## Discussion

Invasive ICP monitoring facilitated by the implantation of a ventricular or parenchymal probe is an acknowledged monitoring tool and is recommended by current guidelines in patients with severe TBI [5]. ICP monitoring gives physicians the opportunity to quickly react to changes in a crucial variable of cerebral perfusion. Despite controversial findings regarding a beneficial outcome for patients under ICP monitoring [12], recent literature suggests improved survival [19].

The aim of the current study was to evaluate the management and clinical decision making in patients under ICP monitoring for severe TBI at a level I trauma center. Special attention was paid to the indication of follow-up CCT scans in the context of ICP monitoring.

We found, to our surprise, that nearly three quarters of follow-up CCT scans were routinely performed in the absence of excessive ICP or clinical features of neurological deterioration. The rate of bleeding progression in these cases was 15.6% compared to beyond 50% when ICP abnormalities or clinical deterioration were present. The ratio of progression to progression-free CCT findings was above 6 in CCT scans performed due to elevated ICP compared to routine CCT studies. Now, one could argue that performing 6–7 CCT scans to detect one bleeding progression seems justifiable. However, our data show that bleeding progression had no consequences in terms of clinical interventions in the majority of cases (38/52, 73.1%, Table 5). Surgical intervention based on progression of CCT findings was required in only 3.1% (6/192, Table 4) of all patients who underwent follow-up CCTs. However, this percentage more than tripled when cases with elevated ICP were considered only. Over 11% (5/45) of follow-up CCTs performed due to increased ICP resulted in a surgical intervention. This finding highlights the utility of ICP monitoring in detecting clinically relevant bleeding progression with the potential need for surgical intervention.

**Table 4:** Proportion of clinical implication per type of indication

	*N* (%)	Aggravation of initial finding (%)	Clinical implication (%)
Routine	137 (64.3)	22 (16.1)	Yes 7 (33.7)
			No 15 (68.2)
ICP elevated	43 (20.2)	24 (55.8)^a^*	Yes 7 (29.2)
			No 17 (62.5)
Clinical deterioration	12 (5.6)	6 (50.0%)*	Yes 0 (0.0)
			No 6 (100.0)

*CCT* cranial computed tomography, *ICP* intracranial pressure

*Significantly elevated as compared to routine (*p* < 0.05)

^a^Two datapoints missing due to incomplete medical records

**Table 5:** Absolute numbers of clinical implications following aggravation of cranial computed tomography (CCT) reading

	Routine	ICP elevated	Clinical deterioration
No consequence	15	15	0
Delay of AT therapy	4	0	0
Craniectomy	0	3	0
Additional contralat. craniectomy	0	1	0
Revision of EDH	1	1	0
Prolonged TBI protocol	1	1	0
Reintubation	1	0	0
Mannitol administration	0	1	0

*AT* anti-thrombotic, *EDH* epidural hematoma, *ICP* intracranial pressure, *TBI* traumatic brain injury

As far as routine follow-up CT scans are concerned, frequent patient transport and transfer to CT units might present an underestimated risk to critically ill patients. Studies demonstrated that intrahospital transport (IHT) can significantly increase ICP in TBI patients [16, 20]. Martin et al. reported a 61% rate of adverse events, defined as ventilator asynchrony, hardware failure, and unintended removal of tubes and lines during IHT of TBI patients [17]. Furthermore, they reported that the incidence of secondary insults was significantly higher during transport as compared to an 8 h interval before and after IHT. Cerebral metabolism, however, seems to remain unaffected by IHT, according to another study by Küchler et al. [21]. Considering the severity of injuries and the lack of data from randomized trials, routinely performing CCT scans still seems legitimate in patients with severe TBI. However, attending physicians should question the clinical relevance and implications, as well as therapeutic consequences of follow-up scans with respect to the risks IHT poses. Especially in the absence of elevated ICP and clinical-neurological deterioration, indications of follow-up CCT scans should be considered with care.

Because bleeding progression on CCT scans without ICP elevation is observable in some cases, the validity of ICP measurement is often questioned during clinical routine. ICP used to be considered constant throughout the cranium. However, studies have demonstrated that ICP values can vary according to the type and site of measurement [22, 23]. As the probe is usually placed contralateral to the site of injury in cases of simultaneous craniectomy, compartmentalization could be a relevant effect resulting in misleading ICP values. In case of craniectomy, the static component of ICP is manipulated, so the overall concept of ICP monitoring must be reconsidered. From this point of view, invasive ICP monitoring must be regarded as an additional diagnostic tool and considered in the overall clinical context. Therefore, as ICP values can be erroneous, this might ultimately be used as argument to justify more liberal indication for performing follow-up scans.

In accordance with the literature, the overall complication rate of ICP monitoring in the present study was 5.6% [15]. While severe complications are rare, bleeding and infection are among the most frequently reported complications associated with invasive intracranial pressure monitoring. Intraparenchymal probes have been associated with a < 1% rate of infection, which is significantly lower than in extraventricular drainage systems [24]. The results of the present study support these findings as there was no case of intra-hospital catheter-associated infection. Overall, there were six bleeding-related complications (Table 3), however, three were subgaleal hematomas without clinical consequences and most likely didn’t affect the outcome. In two cases, the medical records were incomplete, so the exact type of bleeding complication could not be evaluated. There was one case of bleeding along the catheter, which has to be considered a definite catheter-associated complication. Taking into account, the two doubtful bleedings, there were overall three catheter-associated bleeding complications. No major complications were observed. Looking at the present data and the available literature, invasive ICP monitoring can be regarded a safe method in patients with severe TBI. This might also contribute to the fact that ICP monitoring has been incorporated in guidelines regarding TBI. Current innovations in ICP monitoring include telemetric systems that allow data transmission via a reader unit placed on the intact skin [25, 26]. Without the need for a physical connection to the intracranial space, this method is supposed to further decrease complication rates and facilitate long term ICP monitoring even outside the hospital. Although most of the studies were performed in settings of elective neurosurgical procedures, the findings seem promising for an increasing implementation in ICP monitoring following TBI [26].

There are several non-invasive alternatives of ICP-monitoring, including near-infrared spectroscopy, electroencephalography, visual-evoked potentials and retina sonography [27]. The latter has been demonstrated to be an effective diagnostic tool when applied in an appropriate patient collective. In their meta-analysis, Ohle et al. [28] included 12 studies on the diagnostic accuracy of retina sonography in detecting elevated ICP. The results illustrate how this method can be useful in ruling out raised ICP in patients with low pretest probability. Considering that in the present study, follow-up CCT scans were frequently performed in the absence of elevated ICP readings, retina sonography might be an alternative to rule out elevated ICP in these patients. The implementation of this low-cost point-of-care tool might therefore reduce the number of unnecessary CCT scans in TBI patients under ICP monitoring.

The results of the present study demonstrated a high number of follow-up CCT performed in severe TBI patients under invasive ICP-monitoring. In particular, there was a high rate (64.3%) of routinely performed scans despite the absence of elevated ICP readings or clinical/neurological deterioration. The data show that the presence of elevated ICP and/or clinical deterioration is associated with a substantially increased odds ratio for aggravated CCT findings as compared to routinely performed scans. Considering the potential harms caused by frequent patient transport in severe TBI and the availability of additional bedside diagnostic tools, the necessity of routinely performed CCT scans should be evaluated thoroughly.

The authors acknowledge that this study has several limitations. First of all, the retrospective design which relied on the evaluation of ICU medical records. The inherent reporting bias included partly inconsistent records regarding indication for CCT and incomplete information about medication (anticoagulation). Furthermore, the severity and detailed morphology of initial CCT scans was not considered in evaluations. Also, the clinical course following the initial follow-up CCT including further imaging results and consecutive potential interventions were not evaluated due to increased heterogeneity of data. The outcome data were limited by the accuracy of the neurological status as described in transfer reports. Lastly, stratification by CCT indication relied upon the consistency of reporting elevated ICP or clinical deterioration within medical records. Also, post-traumatic intracerebral complications (e.g. hydrocephalus) were not available for review in this study.

## Conclusion

The present data demonstrate a high number of CCT scans performed in severe TBI patients with invasive ICP monitoring. The proportion of progression of initial CCT findings and subsequent clinical implications on routinely performed scans was overall low. Therefore, indications and potential clinical implications should be carefully evaluated in each case.

## Data Availability

The datasets used and/or analysed during the current study are available from the corresponding author on reasonable request.

## Abbreviations

CCT: Cranial computed tomography
CI: Confidence interval
CPP: Cerebral perfusion pressure
CT: Computed tomography
GCS: Glasgow Coma Scale
GOS: Glasgow Outcome Scale
ICP: Intracranial pressure
ICU: Intensive care unit
IHT: Intrahospital transport
IQR: Interquartile range
ISS: Injury Severity Score
MAP: Mean arterial pressure
OR: Odds ratio
RCT: Randomized controlled trial
TBI: Traumatic brain injury
